# Lay understanding and experience of sexual intercourse among couples with infertility undergoing an assisted reproduction technology program: A qualitative study

**DOI:** 10.1016/j.heliyon.2024.e26879

**Published:** 2024-02-22

**Authors:** Cennikon Pakpahan, Agustinus Agustinus, Ashon Sa'adi, Thi Tu An Nguyen, Pranee Liamputtong, Christantie Effendy, Aucky Hinting

**Affiliations:** a Andrology Study Program, Faculty of Medicine, Universitas Airlangga, Surabaya, Indonesia; b Department of Biomedical Sciences, Faculty of Medicine, Universitas Airlangga, Surabaya, Indonesia; c Ferina Mother and Child Hospital, Surabaya, Indonesia; d Department of Obstetrics and Gynecology, Faculty of Medicine, Universitas Airlangga, Surabaya, Indonesia; e Monash University, Melbourne, Australia; f College of Health Sciences, VinUniversity, Gia Lam District, Hanoi, Viet Nam; g Center for Global Health, Perelman School of Medicine, University of Pennsylvania, USA; h Medical Surgical Nursing Department, Faculty of Medicine, Public Health and Nursing. Universitas Gadjah Mada, Indonesia

**Keywords:** Sexual intercourse, Infertility, Assisted reproductive technology, Sexual health, Reproductive healthcare, Well-being

## Abstract

**Aim:**

To explore the perception and lay understanding of sexual intercourse and sexual life experiences among infertile couples before, during, and after undergoing an assisted reproduction technology (ART) program.

**Method:**

The participants of this descriptive qualitative study were Indonesian couples with infertility who underwent an ART program. Semi-structured interviews were conducted between September and December 2022, and the participants' responses were recorded. Data were analyzed using a step-by-step analysis based on Braun's qualitative analysis. The study was reported based on the Consolidated Criteria for Reporting a Qualitative Research (COREQ) Checklist.

**Results:**

Fifty participants were included, and five themes were developed before and two themes during or after the ART program. The couples’ knowledge varied as they experienced sexual intercourse at different periods, such as before, during, and after the ART program. Many participants reported that ART affected their emotions and mood, leading to decreased desire to engage in sexual intercourse. However, some used sexual intercourse as a basis for creating optimism and confidence in having offspring. Furthermore, couples perceived that the purpose of sexual intercourse is not only to have offspring but also to improve communication, promote intimacy, and express affection. In contrast, some perceived the ART program as time consuming, preventing them from engaging in sexual activities. However, not all couples considered sexual activity solely as a means of procreation. They concluded that sexual behavior is not only determined by genetics.

**Conclusion:**

Couples who underwent the ART program regardless of its effectiveness were aware that sexual interaction is not only for having children but also for preserving harmony and familial connection.

## 1 Introduction

Infertility refers to inability to conceive after 12 months or more of regular unprotected sexual intercourse [1]. It occurs due to defects in the female and/or male reproductive system or for unexplained reasons [1]. Individuals with infertility experience high stress levels that may affect their sexual experience as well as quality of life [2]. A complex relationship has been reported to exist between infertility and sexual intercourse [3], with the latter losing its appeal due to this condition [4]. Infertility can lead to stress and fragility in couples [5], and stressful situations can cause a lack of sexual attraction. Studies have reported that couples with long-term infertility struggle with their sexual life [5]. Their efforts to treat infertility by undergoing tests, including sperm analysis (involving masturbation in males) or postcoital tests (in females), occasionally make them realize the disruption in their sexual life [5]. Therefore, infertility is believed to lead to sexual dysfunction [6].

High stress levels have been reported in Indonesian couples, as they experience societal pressure to have children after wedding [7]. Therefore, every couple designs a routine for sexual intercourse that aligns with the ovulation period in attempt to conceive. However, the journey to conception is not always smooth. Infertility diagnosis has negative emotional and psychological effects on couples, culminating in sexual disorders [8]. Indonesians opt for assisted reproductive technology (ART) treatment to deal with the pressure associated with infertility. However, the ART process, involving examination and hormone stimulation, has been reported to test couples’ psychological state, which is linked to their quality of life, including intimacy and sexuality [9].

During the ART process, couples undergo several procedures that sometimes affect their self-image and body acceptance. In a study on inferto-sex syndrome, Luca et al. suggested that failure to conceive causes males to have feelings of masculinity loss, emotional stress, and discomfort. In numerous situations, diagnosis of infertility in males using certain medical terminologies contributes to sexual identity distortion [10]. Additionally, females sometimes experience depression and excessive anxiety, resulting in feelings of guilt and unworthiness as a partner [9]. Hormonal treatment, during which females undergo moods and emotion changes, occasionally alters their sexual behavior [11,12]. These can cause infrequent sexual intercourse due to loss of interest [13]. Moreover, the use of ART involving a third party limits intimacy, and solidity and intimacy between partners are being questioned [9,14,15].

There is an existing assumption that having sexual intercourse while undergoing ART treatment causes the procedure to fail. Courbiere et al. reported that couples who underwent ART treatment in China experienced difficulties with sexual intercourse and opted to pause sexual intercourse for some time [7]. This study aimed to explore the lay understanding and experiences of sexual intercourse among Indonesian couples with infertility undergoing ART treatment.

## 2 Materials and methods

This study adopted a qualitative approach to obtain in-depth knowledge and experience from participants on a sensitive research topic [16]. Data were collected through semi-structured interviews. This study was approved by the Ethics Committee of the Faculty of Medicine of Airlangga University (approval number: 193/EC/KEPK/FKUA/2022).

### 2.1 Samples and settings

The participants included couples who had been undergoing ART at Ferina Hospital, Surabaya, Indonesia. The recruited participants were identified using a purposive sampling method with the following inclusion criteria: undergoing ART program, willing to sign informed consent, able to communicate verbally, and fully aware of the program.

Each participant was interviewed in a one-day care unit after receiving ET. Interviews were conducted by the first author, who was familiar with conducting interviews and counseling patients. Pseudonyms were used to secure data privacy.

### 2.2 Data collection and analysis

Data of 50 couples were collected from September to December 2022 through in-depth interviews that lasted 30–45 min in a quiet private room within the hospital. The interviewer recorded each couple's response. The interviews were conducted in Indonesian and only selected quotes were translated into English. Consent forms were distributed to the participants to sign before the study started. The interviews focused on two main research questions [1]: What are your views and meanings regarding sexual intercourse during ART program, and what is then reflected on before undergoing the program? [2] How is your sexual life now, compared with before ART? A thematic analysis was used to generate themes. The focus of the interviews was to explore each couple's perspective and experience using the above-mentioned questions. The answers depended on the couple's recall, memory, or reflection ability. We did not perform any interventions intervene for the couples. This process followed the six-step guidelines of Braun et al. [17], beginning with familiarizing with the data, generalizing initial codes, searching and reviewing themes, defining themes, and finally writing the theme. The reporting of the study was based on the Consolidated Criteria for Reporting a Qualitative Research (COREQ) Checklist.

### 2.3 Trustworthiness of results

To maintain research rigor throughout the process, we followed several steps [18]. Investigator triangulation and member checking were used [17]. Researchers held regular meetings, discussions, and evaluations to determine questions, process them, and collect data. The interviews were conducted after several consultation meetings with the researcher to build rapport between the interviewer and the interviewee couples. The interview was conducted when the respondent was calm in the room and after the purpose of the study was explained. The interviews were conducted in a private setting to encourage the patients to answer openly without hesitation, considering that the questions we asked were on sensitive topics. The interview results were noted by the interviewer because the patient refuse to record. Each interview result was discussed again with the second and third authors to assess the data quality. The authors were involved in the coding process and decided whether the data were saturated based on the authors’ discussion, and if data saturation had occurred, data collection was stopped.

The number of participants was determined using the saturation theory. This means that data collection continued until few new data could be obtained [16]. The results of the study that had been conducted were discussed again with the lead researcher before they were provided to independent coders (peer debriefing) for analysis. The lead researcher discussed the results with the other authors before settling on the final analysis results.

## 3 Results

### 3.1 Social demographic characteristics

Data of 50 couples were analyzed in this study. Participant characteristics are presented in Table 1. The mean ages of husbands and wives were 37.68 ± 4.33 and 33.88 ± 4.33 years, respectively. The median duration of infertility was 5.65 years (range 1–12 years). Primary infertility was the most common factor observed in this study (65%). Furthermore, the most common cause of infertility was female factor (48%), followed by mixed factor (male and female) and unexplained factors.

**Table 1:** Social demographic characteristics of the participants (n = 50 couples).

Variables		n (%)	Mean ± SD/Median (min-max)
Age	Husband		37.68 ± 4.33 years
	Wife		33.88 ± 4.33 years
Duration of infertility			5.65 [[1], [2], [3], [4], [5], [6], [7], [8], [9], [10], [11], [12]] years
Type of infertility	Primary	39 (65%)	–
	Secondary	11 (35%)	
Religion (Couple)	Islam	33 (66%)	
	Christian	13 (26%)	
	Hinduism	3 (6%)	
	Buddhism	1 (2%)	
Ethnicity	Javanese	30 (60%)	–
	Chinese	9 (18%)	
	Bali	3 (6%)	
	Batak	3 (6%)	
	Banjar	1 (2%)	
	Sundanese	1 (2%)	
	Dayak	1 (2%)	
	Minahasa	1 (2%)	
	Pakistani	1 (2%)	
Etiology/factor of infertility	Female	24 (48%)	–
	Male	8 (16%)	
	Mix	15 (30%)	
	Unexplained	3 (6%)	
Fresh/Frozen ET	Fresh	21 (42%)	–
	Frozen	29 (58%)	

There were no differences in religion and ethnicity between couples; therefore, the results of this study ruled out friction experienced by couples due to differences in these variables. However, further research should be conducted among couples with different religion, ethnicity, and age to further enrich the information related to this topic. Based on the procedures performed in this study, the proportion of those who underwent frozen embryo transfer (ET) was 58%, while that of those who underwent fresh ET was 42%.

The lay understanding of sexual intercourse among Indonesian couples undergoing ART program varied according to period of ART program: before, during, and after.

**Fig. 1:**
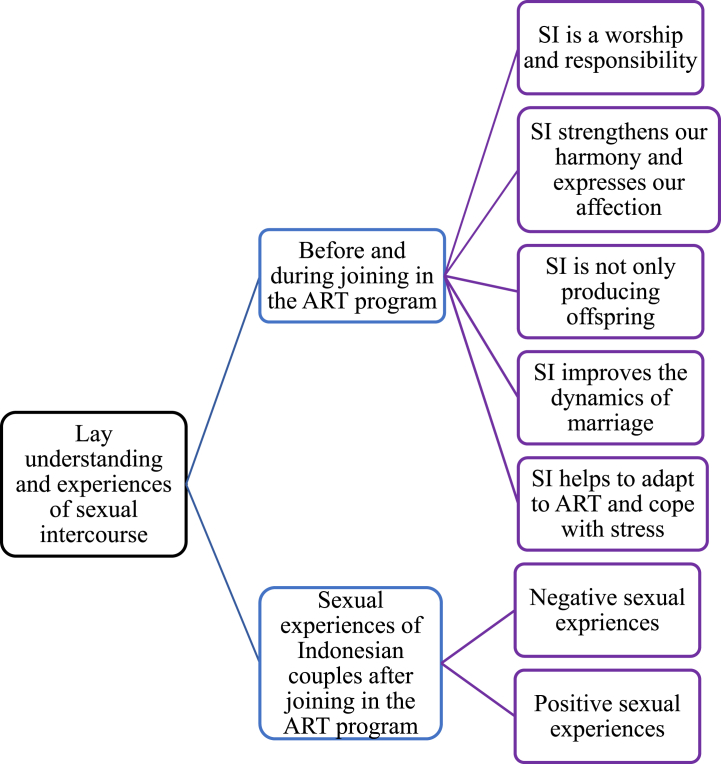
Lay understanding and experiences of sexual intercourse among infertile Indonesian couples.

This study identified five themes of the couples’ perceptions and experiences before and during the ART program: 1). Sexual intercourse is a form of worship and a responsibility, 2). Sexual intercourse strengthens harmony and allows for the expression of affection, 3). Sexual intercourse is not only for producing offspring; it is not everything during marriage, 4). Sexual intercourse improves marriage dynamics, 5). Sexual intercourse helps couples adapt to ART and cope with stress. Two themes were revealed after the ART program: 1). Negative sexual experiences among Indonesian couples in the ART program, and 2). Positive sexual experiences among Indonesian couples in the ART program. This lay understanding of sexual intercourse shaped their negative and positive sexual experiences (Fig. 1).

### 3.2 Lay understanding of sexual intercourse among Indonesian couples before and during the ART program

The role of sexual intercourse in procreation before ART was evaluated. In Indonesia, having children is an expected marital outcome. Thus, Indonesian couples are constantly being asked “Will you have children soon”? This social expectation shapes their sexual perceptions; in turn, they acknowledge that the purpose of sexual intercourse is to have children. According to scientific findings, Indonesian couples frequently perform sexual intercourse for procreation, as they desire to have children after marriage; however, this has been unsuccessful. This failure leads to stress and disappointment.

#### 3.2.1 Sexual intercourse is a form of worship and a responsibility

Some viewed sexual intercourse as a form of worship, not just for procreation. Couples stated that sexual intercourse is God's commandment in marriage. They believe that the motivation to have sexual intercourse is affected by the sole aim of having children; therefore, when they failed to have children naturally, sexual intercourse was not affected.

“… Sex as worship and command from God …” (Couple-32, Primary, Frozen ET, 34-year-old male)

*“Sexual intercourse is not only to get offspring but also part of worship*” (Couple-13, Primary, Fresh ET, 32-year-old female).

Apart from being a form of worship, sexual intercourse was also reported to be a marital responsibility between a couple. When sexual intercourse was considered a responsibility, it was not postponed, even when the couple failed to conceive naturally or were in the ART program.

*“… But I still do it as a form of responsibility as a wife”* (Couple-12, Secondary, Frozen ET, 34-year-old female).

The perspective of sex as a form of worship was expressed by couples who received fresh ET and frozen ET, as well as in those with primary and secondary infertility.

#### 3.2.2 Sexual intercourse strengthens our harmony and helps us to express affection

The ART process left couples in a hectic state accompanied by anxiety, as they worried about the program's success. Couples undergoing ART admitted that sexual intercourse helped them to maintain harmony in their relationship, especially during difficult times in the ART program. The feeling of intimacy is created through sexual intercourse, through which they shared their feelings and desires, including stories. They realized that this was an excellent start to strengthening their relationship.

*“For us, having sexual intercourse is not only because we want to have children but also because we can enjoy each other to get closer*” (Couple-22, Secondary, Frozen ET, 30-year-old male).

*“We know having sexual intercourse helps us maintain an intimate interaction between the two of us”* (Couple-26, Secondary, Frozen ET, 32-year-old female).

*“As a husband, I feel that having sex is a form of love and affection for my wife*” (Couple-12, Secondary, Frozen ET, 35-year-old male).

*“We are currently in a long-distance marriage, we are not in regular intercourse due to circumstances. We are currently in a program, but we are aware that sex is useful to maintain the harmony of our household*” (Couple 14, Primary, Fresh ET, 30-year old female).

In addition, couples believed that sexual intercourse was a way of showing affection for their partners to increase harmony. Sexual intercourse concerned how they expressed their love for their partners, particularly during the ART program.

*“The sexual intercourse between us is currently good, we feel happy with our current sexual intercourse life. We are conscious of making our relationship feel more intimate, this is our way of showing the expression of affection between us”* (Couple-2, Secondary, Fresh ET, 33-year-old female).

*“… For me, sex helps me maintain feelings and bonding (chemistry) with my wife. This is my affectionate language towards my wife*” (Couple-23, Secondary, Frozen ET, 30-year-old male).

#### 3.2.3 Sexual intercourse is not only for producing offspring; it is not everything in marriage

Some couples had a broad perspective regarding sexual intercourse during marriage, particularly when undergoing ART. Couples realized that sexual intercourse is necessary but is not everything in marriage. For them, sexual intercourse is not just an activity performed for procreation; therefore, changes resulting from failing to have children and subsequently undergoing ART did not change the basic principles of sexual intercourse. They persisted in having sexual intercourse, not solely to have children but because it was necessary.

*“Thankfully, we still maintain the quality of our relationship because sexual intercourse is important, but not everything. So, after that, nothing was different about sexual intercourse between us”* (Couple-29, Secondary, Frozen ET, 33-year-old female).

*“… Sex for us is not just about having children”* (Couple-42, Primary, Fresh ET, 38-year-old male).

Sexual intercourse improves the dynamics of marriage.

Although the couples had failed to have children naturally and were required to spend extra money and effort on ART, sexual intercourse was considered to provide several benefits. One of the benefits of engaging in sexual intercourse while undergoing ART was emotional stability. Although ART program caused mood and emotion disturbance, couples reported that sexual intercourse helped them maintain a balance.

*“Sexual intercourse is undoubtedly related to emotions between us, so sexual intercourse must still be a way to maintain feelings between us”* (Couple-33, Primary, Frozen ET, 31-year-old female).

Couples also realized that sexual intercourse was one of the activities that helped them feel complete as husband and wife. Sexual intercourse is an intimate activity between partners that involves physical, psychological, and spiritual needs.

“*Having sexual intercourse is the most intimate time together to meet our physical, psychological, and spiritual needs*” (Couple-11, Secondary, Frozen ET, 32-year-old female).

Another interesting aspect expressed by couples undergoing ART was that sexual intercourse provides benefits by influencing the marriage dynamics. They realized that sexual intercourse created an avenue for intimacy and harmony; therefore, sexual intercourse improved the dynamics of their relationship.

*“Sexual intercourse does influence the course of the dynamic family in our relation”* (Couple-35, Secondary, Frozen ET, 44-year-old male).

Furthermore, the benefits they experienced include better marriage quality owing to frequent sexual intercourse. The principle is that couples understand that sexual intercourse affects the entire marriage. Moreover, they stated that sexual intercourse was infrequent during marriage, indicating the presence of a problem in the relationship.

*“We realize that the quality of sexual intercourse will affect the quality of our marriage, so we take care of our sexual intercourse life, including during this program*.” (Couple-43, Primary, Fresh ET, 29-year-old female).

#### 3.2.4 Sexual intercourse helps to adapt to ART and cope with stress

Sexual intercourse during ART enabled couples to adapt to the program they were undergoing and used sexual intercourse as a stress-coping mechanism while undergoing the program.

*“Sexual intercourse helps me relieve stress a lot. I feel a lot tired and bored right now. Sexual intercourse helps me more relax*” (Couple-50, Primary, Frozen ET, 39-year-old female).

Additionally, other couples believed that sexual intercourse refreshed them when they were tired during the program.

*“Sexual intercourse is indeed a time for us to refresh, a time to show affection between us, we understand this* …” (Couple-25, Primary, Frozen ET, 43-year-old male).

Before undergoing ART treatment, couples had different perceptions of sexual intercourse. Some argued that sexual intercourse is a form of worship or a marital responsibility. However, while undergoing ART treatment, their perception of sexual intercourse improved. They agreed that sexual intercourse is not only about having children but also about responsibility, worship, and maintaining family unity and harmony, as well as maintaining emotional bonds as a couple. Even during their ART journey, some couples admitted they use sexual intercourse as a stress-coping mechanism in their current situation.

### 3.3 Sexual experiences of Indonesian couples after joining the ART program

The participants in this study reported both negative and positive sexual experiences after joining the ART program. The details are presented below.

#### 3.3.1 Negative sexual experiences among Indonesian couple undergoing ART

Participants undergoing ART expressed their views on how the treatment has impacted their sexual life. They described their struggles to maintain a relationship through sexual activity. Many of their concerns were related to sexual intercourse during ART. Various studies have reported the relationship between ART and sexual intercourse. This theme included participants’ responses regarding the influence of ART on their sexual life, their sexual life before undergoing ART, and while undergoing ART.

#### 3.3.2 ART decreases sexual intercourse frequency

Couples undergoing ART reported a decrease in the frequency of sexual intercourse due to the program. ART changed their sexual intercourse habits due to hormonal factors, mood changes, or their own anxiety. Furthermore, they realized that, during the ART program, they felt something was missing in their relationship but could not explain it. This also caused the decrease in sexual intercourse frequency.

*“… it makes it feel like something is missing in our relationship, which used to be in sexual intercourse regularly but now not at all during the program”* (Couple-36, Secondary, Frozen ET, 31-year-old female).

Before undergoing ART, some couples rarely had sexual intercourse, and undergoing the ART program further made them less likely to have sexual intercourse.

*“At this time, we rarely have sexual intercourse because we adapt to the program being undertaken* …” (Couple-50, Primary, Frozen ET, 39-year-old female).

#### 3.3.3 ART affects our emotions

The intense ART process keeps couples trapped and affects their emotions. ART, which is expensive, also makes them careful about their actions. ART made couples highly sensitive to certain things and frightened them to thinking even the slightest action would make the program unsuccessful.

*“Right Now, during the program we are afraid to have sexual intercourse, we afraid it will affect the program*” (Couple-15, Primary, Fresh ET, 32-year-old female).

*“… However, to be honest, if this program fails, you don't know what it will be like …”* (laugh) (Couple-11, Secondary, Frozen ET, 32-year-old male).

During these times, the ART process caused emotional instability, as well as depression and anxiety. Their emotions were examined during ART. They admitted that this decreased their desire and passion, thereby affecting their interest in sexual intercourse. Additionally, they occasionally forced themselves to have sexual intercourse; however, the results were not as expected.

*“During the ART program, sometimes, we did not enjoy our sexual intercourse, and sometimes I felt bored with the current conditions*” (Couple-16, Primary, Frozen ET, 36-year-old female).

*“… The ART program put us under pressure, affected our mood, and we even felt compelled to have sex*” (Couple-28, Secondary, Fresh ET, 30-year-old male).

#### 3.3.4 Positive sexual experiences among Indonesian couple undergoing ART program

Participants also shared that ART positively affected their sexual life. Couples began to form a new understanding of satisfaction without the need for penetration. This unique experience helped them to remain intimate while undergoing ART. Moreover, ART improved their chemistry.

##### 3.3.4.1 Feeling pleasure without penetration

For some couples, satisfaction or intimacy does not always have to lead to sexual intercourse, in which vaginal penetration occurs; however, it could be derived from other activities, including cuddling time, deep talk, or pillow talk. ART made partners very careful about activities that lead to penile penetration into the vagina. They were afraid that penetration would cause the program to fail. Some couples believed that spending quality time together without sexual intercourse was sufficient for achieving pleasure. Couple time without penetrative sexual intercourse was not absolutely for them to experience pleasure.

*“For us, having an intimate time alone (deep talk, sharing) without having to have sexual intercourse is satisfying for both of us. Intimacy and satisfaction in our marriage doesn't always have to be sexual intercourse with the penetration of the penis into the vagina*” (Couple-36, Secondary, Frozen ET, 31-year-old female).

##### 3.3.4.2 ART helps to improve chemistry through sexual intercourse

ART does not permanently cause harm to couples' lives. It does not cause emotional instability that leads to anxiety and disinterest in sexual intercourse. ART, one of the stages of a couple's struggle to have offspring, requires commitment and cooperation. Couples admitted that sexual intercourse improved their chemistry and prepared them better for ART. This also made them unhesitant to have sexual intercourse during the program.

*“Currently, when we are already in the ART program, the quality of our relationship is much better. We enjoy having sexual intercourse, so we do more sexual intercourse”* (Couple-49, Primary, Frozen ET, 30-year-old male).

*“… Our sexual life are still going well. We still have sex even before the embryo transfer”* (Couple-32, Primary, Frozen ET, 31-year-old male).

Overall, our thematic findings did not differ significantly between couples with primary and secondary infertility. The same applies to couples who underwent fresh ET and frozen ET procedures. We did not analyze these groups separately; however, based on our observations, a few themes were expressed more in the primary infertility group than the secondary infertility group. For example, “ART helps to improve chemistry through sexual intercourse” and “sexual intercourse helps to adapt to ART and cope with stress.” This suggests that ART has a positive impact on the sexual life of couples with primary infertility.

## 4 Discussion

This study contributes to the knowledge of the lay understanding and experience of sexual intercourse among couples undergoing ART in Indonesia. The strenuous ART process affects couples’ sexual life. Previous quantitative studies have reported a marked decrease in sexual activity in couples undergoing ART [6]. However, to the best of our knowledge, this is the first qualitative study on this topic.

Our findings showed that sexual intercourse is not always influenced by ART. The dominant partner was observed to be relatively dissatisfied with their sexual life during ART. This study showed that some couples still experience good sexual activities; they attempt to have regular sexual intercourse when they are relaxed; however, the issue of mood changes is still being considered. Several reasons underlie this behavior, one of which is the type of partner infertility. Couples with prior children are not burdened by ART. This is consistent with a study by Epstein and Rosenberg [19], who reported that secondary infertility leads to a milder level of depression than primary infertility, whereas some previous studies have reported that depression affect couples’ sexual life. Furthermore, some couples believe that ART and sexual intercourse are not related. Afshani et al. suggested that a positive attitude toward ART is needed to for couples to make the right decisions [20].

Willingness to continue sexual intercourse was also observed in couples who committed to having regular sexual intercourse before the ART program. Their decision to have regular sexual intercourse is not only because they desire to have children but also because, from the start, their perception of sexual intercourse has been fixative. Some couples had not had children for 12 years; however, they remained committed to sexual intercourse. Baczkowski et al. stated that infertility duration does not significantly affect a couple's sexual intercourse [21]. Furthermore, it encourages couples to realize that the failure of the ART program is not an obstacle to not having sexual intercourse [21]. Moreover, they reported that not all couples undergoing ART experienced decreased sexual activity. Only 7.1% reported a significant decline in their partner's interests [21]. Conversely, couples who previously had a poor sexual life remained unsatisfied during the ART program.

However, our study also showed that partners experienced degradation during sexual intercourse because of emotional changes. Similar findings have been reported I previous studies [14,22]. ART affects partner emotions, quality of relationships, and sexual life. The participants stated that ART puts them under pressure, thereby affecting their moods and emotions. Couples are preoccupied with examinations and various procedures to increase their likelihood of conceiving, which causes anxiety, fright, and boredom. Therefore, they often avoid sexual intercourse. Couples experience this mixed feeling, leading to degradation of both the quality and quantity of sexual intercourse.

The Indonesian couples in this study also reported experiencing saturation during sexual intercourse. In some relationships, sexual intercourse loses its appeal. Millheiser et al. reported a similar decrease in sexual intercourse arousal and frequency, thereby reducing the frequency of intercourse [23]. This trend tended to be observed in females. Additionally, the reason for the duration of the desire to have children also causes couples to feel tired and uninterested in their relationship and has an impact on sexual activity [24]. Couples experience challenging during ART programs; therefore, it is sometimes emotionally draining to use sexual intercourse as an alternative for stress relief. Even couples admitted that sexual intercourse isa coping mechanism for anxiety, particularly when there is pressure during the ART program. These results are reasonable and expected, considering that sexual intercourse leads to the production of oxytocin, dopamine, and endorphins, which trigger relaxation [25]. Yoo et al. state that couples who are usually satisfied with sexual intercourse are also relatively emotionally stable [26]. Furthermore, couples are afraid of sexual intercourse, because they believe that it will affect the program and that sexual intercourse is harmful to women and decreases their likelihood of having offspring. However, studies have reported that sexual intercourse during ART programs, particularly before ET, has a positive effect on patients [27].

Additionally, we observed that the participants’ age was one of the reasons why they perceived sexual intercourse as sometimes unattractive, in addition to the busyness factor between partners. Muller et al. reported that age and infertility duration frequently are factors that affect interest in sexual intercourse [28]. Age may also be associated with hormonal changes [29]. Interestingly, none of the couples participants of this study reported erectile dysfunction or premature ejaculation in males or sexual intercourse dysfunction in females.

Several participants of this study admitted that ART provided them with confidence about having children. Considerable optimism and positive energy have also been reported in a previous study [22]. However, another study reported that infertility decreases self-confidence [30]. Confidence may be influenced by the attitude and knowledge of a partner, and this can change the stigmatization associated with ART, which is synonymous with failure, to an opportunity. This is consistent with a finding that education can increase self-confidence through a good understanding of the problem [31].

In this study, we observed a positive finding that a partner's perspective on sexual intercourse is not constantly flawed when undergoing ART. Couples recognized that sexual intercourse is not just an activity for procreation to maintain the continuity of a family institution. With the strong religious culture and values of Indonesians, couples persisted with having sexual intercourse despite not necessarily becoming pregnant. They consistently engaged in sexual intercourse, believing that it is part of their worship. Understanding this leads them to the commitment to having sexual intercourse as their marital responsibility. This is consistent with the finding of a study that stated that understanding religious values makes a person engage in sexual intercourse [32]. Sexual intercourse is necessary for married couples and is vital in household relationships. Having such positive attitude helps partners to commit to maintaining their sexual life, regardless of the success of ART treatment. However, partners must be careful with this concept because it may cause feelings of guilt if not sexual intercourse is not performed [32].

Couples realize sexual intercourse not only helps to procreate but also to stay harmonious and intimate, maintain the quality of marriage, and provide a dynamic feeling for the family. Sexual intercourse is used to express between partners. It is important to understand the perspective of couples with regard to maintaining their sexual life, despite experiencing pressure and emotion change related to ART treatment. Similar to the study reported by McNulty et al., couples felt that relationship satisfaction bidirectionally affected the quality and frequency of sexual intercourse [33].

Sexual intercourse, identified as penile penetration into the vagina, is not a fixative and rigid idea in couples’ lives. Penetration does not often occur because the effort to reach that point is not always present or lasting. However, other ways of enjoying each other or showing affection without penetration can be explored. One of such alternatives is a concept known as “sensate focus.” [34]. This concept enables couples to maintain intimacy without penetration while undergoing ART treatment.

Although this study provides broad and varied perspectives on sexual intercourse among couples undergoing ART, some limitations were observed. First, the participants were not followed-up after the study. Performing a follow-up regarding ART will be helpful. Moreover, combining quantitative and qualitative methods (mixed-method study) has become a richer approach for interpreting any sexual intercourse-related findings in couples undergoing ART treatment. Second, there may have been cases of misinformation. We tried to conduct a full interview when both wife and husband were present, but in some couples, we obtained more quotes/perspectives from either of the partners, assuming that the recorded perspectives were based on the agreement of both partners. Some couples added to each other's answers, whereas others only agreed with their partners' answers. Joint answers were assumed to be joint opinions. However, this could be explored further by conducting separate interviews among couples because there may be reluctance to express opinions freely in the presence of their partners even though they are married.

Finally, we did not assess differences in ethnicity or religion between partners, which may have provided a broader perspective on this topic; therefore, we propose further research on interracial and interfaith couples. In addition, we did not assess differences between couples who received fresh ET and those who received frozen ET. These two procedures may provide different reflections and perspectives on this matter.

## Data availability statement

Supplementary data to this article can be found online at https://doi.org/10.6084/m9.figshare.24989733.v1.

## CRediT authorship contribution statement

**Cennikon Pakpahan:** Writing – review & editing, Writing – original draft, Validation, Project administration, Methodology, Formal analysis, Data curation, Conceptualization. **Agustinus Agustinus:** Writing – review & editing, Writing – original draft, Methodology, Investigation, Data curation, Conceptualization. **Ashon Sa'adi:** Writing – review & editing, Supervision, Investigation, Formal analysis, Conceptualization. **Thi Tu An Nguyen:** Writing – review & editing, Writing – original draft, Visualization, Validation. **Pranee Liamputtong:** Writing – review & editing, Writing – original draft, Validation, Supervision. **Christantie Effendy:** Writing – review & editing, Writing – original draft, Validation, Supervision. **Aucky Hinting:** Writing – review & editing, Supervision, Methodology, Investigation.

## Declaration of competing interest

The authors declare that they have no known competing financial interests or personal relationships that could have appeared to influence the work reported in this paper.
